# Proteomic identification of fucosylated haptoglobin alpha isoforms in ascitic fluids and its localization in ovarian carcinoma tissues from Mexican patients

**DOI:** 10.1186/1757-2215-7-27

**Published:** 2014-02-27

**Authors:** Olga Lilia Garibay-Cerdenares, Verónica Ivonne Hernández-Ramírez, Juan Carlos Osorio-Trujillo, Magdalena Hernández-Ortíz, Dolores Gallardo-Rincón, David Cantú de León, Sergio Encarnación-Guevara, Julio César Villegas-Pineda, Patricia Talamás-Rohana

**Affiliations:** 1 Departamento de Infectómica y Patogénesis Molecular, Centro de Investigación y de Estudios Avanzados del Instituto Politécnico Nacional, Av. Instituto Politécnico Nacional 2508, Col. San Pedro Zacatenco, Delegación Gustavo A. Madero, México, D.F. 07360, México; 2 Instituto Nacional de Cancerología, SSA, México, D.F. Av. San Fernando 22, Sección 16, Tlalpan, México, DF 14080, México; 3 Centro de Ciencias Genómicas, Universidad Nacional Autónoma de México, Av. Universidad s/n Col. Chamilpa, Cuernavaca, Mor 62210, México

**Keywords:** Ascitic fluid, Fucosylation, Haptoglobin, Ovarian carcinoma, Proteomics

## Abstract

**Background:**

Ovarian cancer is the most lethal gynecologic disease due to delayed diagnosis, and ascites production is a characteristic of patients in advanced stages. The aim of this study was to perform the proteomic analysis of ascitic fluids of Mexican patients with ovarian carcinoma, in order to detect proteins with a differential expression pattern in the continuing search to identify biomarkers for this disease.

**Methods:**

Samples were collected from 50 patients from the Instituto Nacional de Cancerología of México under informed consent and with approval of the bioethics and scientific committees. After elimination of abundant proteins (Albumin/IgGs) samples were processed for 2D electrophoresis and further protein identification by Mass Spectrometry (MALDI-TOF). Molecules of interest were followed by western blot and lectin binding assays, and their tissue location by histo-immunofluorescence and confocal analysis.

**Results and discussion:**

An area with a differential expression pattern among samples was located in the 2D gels. Identified proteins were 6 alpha 1 isoforms and 1 alpha 2 isoform of Haptoglobin, and 2 isoforms of Transthyretin. While Transthyretin isoforms were constitutively expressed in all samples, clear differences in the expression pattern of Haptoglobin alpha isoforms were found. Moreover, increased levels of fucosylation of Haptoglobin alpha isoforms analyzed in 40 samples by *Aleuria aurantia* lectin binding by 1D overlay assay showed a positive correlation with advanced stages of the disease. Tissue detection of Haptoglobin and its fucosylated form, by histo-immunofluorescence in biopsies of ovarian cancer, also showed a correlation with ovarian cancer progression. Moreover, results show that fucosylated Haptoglobin is produced by tumor cells.

**Conclusions:**

Increased numbers of highly fucosylated Haptoglobin alpha isoforms in ascitic fluids and the presence of fucosylated Haptoglobin in tumor tissues of ovarian cancer Mexican patients associated with advanced stages of the disease, reinforce the potential of fucosylated Haptoglobin alpha isoforms to be characterized as biomarkers for disease progression.

## Background

Epithelial ovarian cancer (ovarian carcinoma) is the most lethal gynecologic malignancy worldwide [1]. In the latest data recorded by GLOBOCAN compiled in 2008, Mexico reported an annual incidence of 2,910 cases and a mortality of 1,851 Mexican women during that year (http://globocan.iarc.fr). According to the Mexican National Cancer Institute, ovarian cancer is at the fifth position among the 10 most frequent causes of hospital mortality during 2012 (http://www.incan.edu.mx/). The high mortality rate is attributable to the asymptomatic nature of the early stage of the disease, the lack of established screening tests, and the development of drug resistance [2-5].

Like most other epithelial tumors, ovarian carcinoma spreads initially into adjacent organs, specially the fallopian tubes and uterus. It then disseminates via the transcoelomic route, affecting many vital organs within the abdomen including the gastrointestinal and genitourinary systems [6,7]. Over 30% of patients present accumulation of peritoneal fluid or ascites as a sign associated with advanced stages of the disease; and the shedding of malignant cells from the surface of the ovarian tumor into the peritoneal cavity is a common scenario in the progression of ovarian carcinoma [6,8].

Body fluids constitute an excellent media for biomarker discovery, and ascitic fluids usually contain malignant epithelial cells and activated mesothelial cells, which can produce cytokines, growth factors, and invasion-promoting components associated with invasion and metastasis [9,10]; this fluid therefore, contains the secretome of ovarian cancer cells and the analysis of ascites may facilitate discovery of more sensitive and specific biomarkers than those currently available [11].

Several authors have performed proteomic studies in ascites and tumors from ovarian patients describing new biomarkers; among them, E-cadherin and IL-8 as single biomarkers [12,13] or multimarker as CA-125 in combination with macrophage chemotactic protein-1, with leptin, with HE4 or with soluble mesothelin related protein [14-16] have been described. They possess acceptable characteristics for their possible use in population screening, to differentiate among benign and malignant tumors versus healthy controls, and to be used as monitoring biomarkers during cancer progression [17,18].

In this study, we present a proteomic analysis of 50 human ascitic fluids from ovarian carcinoma patients and whose results show, for the first time, clear differences in the profile of Haptoglobin (Hp) alpha isoforms, the presence of genotypes, and the differential fucosylation pattern of the same isoform among samples. Moreover, the presence of fucosylated Hp was confirmed in pathology samples of the same cases. Results suggest an association between the Hp alpha subunit and the fucosylation level with advanced stage of the disease.

## Methods

### Patients

The study was conducted with ascitic fluids obtained from 50 patients diagnosed with ovarian carcinoma who were admitted to the Instituto Nacional de Cancerología de México. All patients were admitted for a first-time diagnosis; the histopathology and tumor grade were assigned by a pathologist according to the International Federation of Gynecology and Obstetrics (FIGO) criteria [19]. The study was approved by the Institutional Scientific and Bioethics Committees (protocol numbers INCAN/CC/134/09 and CB/549/09), and written consent was obtained from patients prior to sample collection.

### Samples

Ascitic fluids samples from patients with ovarian carcinoma were obtained under sterile conditions by a qualified medical doctor. Once collected, they were transported to the laboratory in an ice bucket. Samples were centrifuged at 1500 × *g* for 10 min at 4°C. Supernatants were stored at −70°C until assayed.

### Two-dimensional (2D) gel electrophoresis of ascitic proteins

High-abundance proteins (albumin and IgG) were removed from the ascitic fluid using Vivapure anti HSA/IgG for Human albumin and an IgG depletion kit (Sartorius Stedim Biotech, Cat. No. VS-P08HAIGG), according to the manufacturer’s protocol. Ascites fluids depleted from HSA/IgG (200 μg) were cleared of salts contamination with a ProteoExtract Protein Precipitation Kit (Calbiochem, Cat. No. 539180) as per manufacturer’s protocol. Pellets were processed as described [20]. Briefly, samples were diluted in rehydration buffer containing 8 M urea, 0.5% (w/v) CHAPS, 10 mM DTT, 0.001% bromophenol blue, and Bio-Lyte 3–10 Ampholyte (0.2%) (Bio-Rad, Cat. No.163-1113). The protein mixture was then applied to ReadyStrip™ IPG 7 cm strips, pH 5–8 (linear) (Bio-Rad, Cat. No. 163–2004). Rehydrated strips were isoelectrically focused using a PROTEAN IEF cell System (Bio-Rad, Cat. No. 165–4000). To perform the second dimension analysis, the strips were processed by 15% SDS-PAGE and a Protean II XL Cell System was used. Finally the 2D gels were stained with Silver Stain Plus (Bio-Rad, Cat. No. 161–0449).

### Protein identification by MALDI-TOF

Silver-stained 2D gels were scanned in a GS-800 densitometer (Bio-Rad, Hercules, CA). Digital images were compared using the Melanie 7.0 software (GE Healthcare). Each of the 50 ascitic fluid samples was run three times. The electrophoretic entities of interest were excised, alkylated, reduced, digested in a Proteineer line (Bruker Daltonics, Bremen) with the aid of a DP Chemicals 96 gel digestion kit (Bruker Daltonics) and processed by a MALDI-TOF Autoflex (Bruker Daltonics) to obtain a peptide mass fingerprint. Peak lists of the tryptic peptide masses were generated using FlexAnalysis1.2vSD1 Patch 2 (Bruker Daltonics) [21]. The search engine MASCOT server 2.0 was used to compare the fingerprints against human taxonomy with the following parameters: one missed cleavage allowed, carbamidomethyl cysteine as the fixed modification and oxidation of methionine as the variable modification. Proteins with scores greater than 50 and a p < 0.05 were accepted.

### Western blot and lectin binding assays

Proteins from the ascitic fluid samples (50 μg) were separated either by a 15% or 12.5% SDS-PAGE and transferred to nitrocellulose membranes for subsequent detection of Hp and fucosylated-Hp. Filters were blocked with TBS-T-5% milk, and incubated with anti-Hp antibody (Abcam, Cat. No. ab90924), followed in the case of the 15% SDS-PAGE by a goat anti-mouse IgG-Alkaline Phospatase conjugated antibody (PIERCE, Cat. No. 31320), and in the case of the 12.5% SDS-PAGE by a goat anti-mouse IgG HRP-conjugated antibody (PIERCE, Cat. No. 31430). The filter was then revealed by chemoluminescence (Supersignal West Femto Luminol, Thermo Scientific, Cat. No. 1856189). Duplicate filters were used for fucosylation analysis. Biotinylated *Aleuria aurantia* lectin (AAL) (Vector Labs, Cat. No. B-1395) purified from mushrooms [22] was selected to specifically detect fucosylation of Hp alpha due to its ability to bind preferentially to fucose-linked (α -1,6) to *N*-acetylglucosamine structures (glycosylation’s core). The membranes were incubated in PBS containing approximately 20 μg/ml AAL. Following this, the membrane containing the samples from the 15% SDS-PAGE was incubated with alkaline phosphatase-Streptavidin (Vector Labs, Cat. No. SA-5100), and with the substrate for alkaline phosphatase, BCIP/NBT (Cat. No. SK-5400). The membrane containing the samples from the 12.5% SDS-PAGE was incubated with HPR-streptavidin (Sigma Aldrich, Cat. No. S5512) and developed by chemoluminescence (Supersignal West Femto Luminol, Thermo Scientific, Cat. No. 1856189). The level of fucosylation of Hp alpha was obtained by densitometric analysis (GS-800 Calibrated Densitometer, Bio-Rad) using the Quantity One software for PC (Bio-Rad).

### Detection of fucosylated Hp alpha in ovarian carcinoma tissues by confocal microscopy

Paraffin-fixed biopsies from four ovarian cancer patients and one sample of cancer-free ovarian tissue removed from a patient diagnosed with endometriosis were assessed using a standard histo-immunofluorescence method. Fucosylated-Hp was detected by co-localization of Hp with a primary anti-haptoglobin antibody as described above, and a secondary TRITC-conjugated antibody (goat anti-mouse IgG-TRITC conjugated, Sigma Aldrich, Cat. No. A16077). Fucosylation levels were detected with biotinylated AAL and visualized with Streptavidin-FITC (Life Technologies, Cat. No. 43–4311). Cell nuclei were stained with 4′, 6-diamidino-2-phenylindole dihydrochloride (DAPI) (Sigma Chemical Co., St. Louis, MO, USA). Tissue sections incubated with secondary reagents (antibody or streptavidin alone) served as negative controls. Fluorescent images (10X: tile scan of 5 x 5, 40X, and 63X) were obtained using a Confocal Carl Zeiss LMS 700 microscope that operates with up to four stable, solid-state lasers and wavelengths of 405 to 639 nm. Images were analyzed with the Zen 2011 ‘blue edition’ software. Fluorochromes used were DAPI (4′, 6-diamidino-2-phenylindole dihydrochloride) with excitation and emission wavelengths of 350 and 470 nm, respectively; Fluorescein isothiocyanate (FITC), with excitation and emission wavelengths of 490 and 525 nm, respectively; and Tetramethyl Rhodamine Isothiocyanate (TRITC) with excitation and emission wavelengths of 557 and 576 nm, respectively.

### Statistical analyses

Statistical analyses were performed using STATA v. 12.0 for Windows 2010. A scatter plot graphic was used to show the relationship between two groups of data: the number of detected proteins by 2D electrophoresis of each sample, versus the clinical stage of each patient. The frequency of different genotypes of alpha isoforms of Hp was determined by producing a histogram of frequencies among the tested population. To determine the relation between fucosylation and clinical stage, a Spearman’s rank correlation coefficient was obtained and results were considered significant with a p ≤ 0.05. Finally, a stacked histogram plot was created to visualize the distribution of the fucosylation level of Hp alpha with respect to the clinical stage of the illness.

## Results

After reviewing clinical records of 50 patients, we were able to guarantee the presence of the 5 histologies of the epithelial ovarian cancer, with a higher incidence of the papillary serous type which corresponds with data reported worldwide (http://www.ovariancancer.org/); all the patients with ovarian carcinoma selected for this study produced ascitic fluid. The average age was 52 years, 10 years less than that reported by the American Cancer Society (http://www.cancer.org/). Table 1 shows the family history data associated with ovarian, breast and colorectal cancer.

**Table 1:** Clinical data of ovarian carcinoma patients

**Characteristics**		**Percentage ( *n *)**
**Histopathologic types**	Papillary serous	74 (37)
	Mucinous	12 (6)
	Endometrioid	6 (3)
	Clear cells	4 (2)
	Poorly differentiated	4 (2)
**Clinical stage**	IIB	4 (2)
**(FIGO stage)**	IIIC	52 (26)
	IV	44 (22)
**Age**	20-40	22 (11)
	41–60	56 (28)
	61-80	22 (11)
**Family history of cancer**	YES	26 (13)
	NO	74 (37)

**Figure 1:**
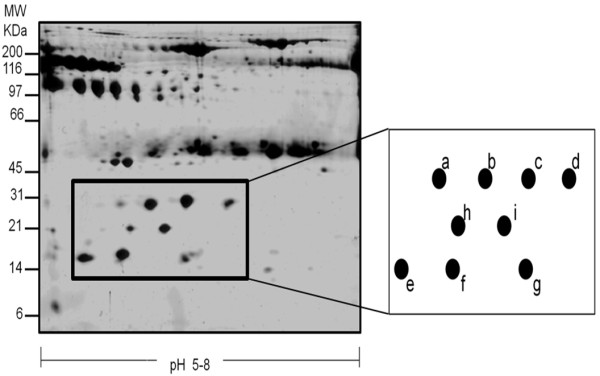
**Detection and selection of a differential expression profile zone.** Silver staining pattern of a representative 15% SDS-PAGE of an ascitic fluid sample with more than 250 proteins resolved, after elimination of abundant proteins; an area with a differential expression profile showing nine protein spots, present in 100% of samples, was selected (black square); schematic representation of the differential expression area and a closer view of the resolution of the corresponding spots (a-g).

Analysis of differential expression of proteins in body fluids such as ascitic fluid allows the detection of proteins involved in different processes, such as proliferation and metastasis. In this study, we show the proteomic analysis of ascitic fluids from patients with ovarian carcinoma. Figure 1 shows the protein profile used as a reference pattern after the elimination of abundant proteins. This elimination process allowed the visualization of more than 250 spots that were resolved for each 2D gel of the 50 samples analyzed.

A zone showing a differential protein expression pattern (Figure 1, black square) was selected, where it was possible to distinguish several spots in every sample. Inside this square as many as nine spots, with highly distinctive expression patterns were detected. To compare samples, Melanie 7.0 software (http://world-2dpage.expasy.org/melanie/) was used, which is a platform that allows the qualitative and quantitative evaluation of protein profiles from bidimensional electrophoresis gels (Additional file 1: Figure S1A). To perform large-scale comparative studies, the software eliminates variations between experiments and detects differential expression profiles. These differential expression profiles were validated using densitometric analysis of the selected area, where notable variations in at least seven of the nine spots were observed; whereas the last two spots showed a similar behavior (Additional file 1: Figure S1B).

The profile of proteins achieved under these conditions was reproducible among samples, and the identification of the nine spots was achieved by Mass Spectrometry (MALDI-TOF). Results indicated that seven spots corresponded to Hp alpha 1 isoforms (a-g) and the remaining two spots (h and i) corresponded to isoforms of Transthyretin (Table 2).

**Table 2:** Protein identification by mass spectrometry (MALDI/TOF)

**Spot ID**	**Protein name**	**GI NUMBER ( *NCBI search *)**	**Theoric**		**Experimental**		**Score**	***e***
			***Mr***	***pI***	***Mr***	***pI***		
**1**	Haptoglobin isoform 1 preproprotein [*Homo sapiens*]	gi|4826762	45861	6.13	28.9	6.4	86	0.0012
**2**	hp2-alpha [*Homo sapiens*]	gi|296653	42126	6.25	28.9	6.2	111	9.5e-05
**3**	Haptoglobin isoform 1 preproprotein [*Homo sapiens*]	gi|4826762	45861	6.13	28.9	6.6	87	0.00097
**4**	Haptoglobin isoform 1 preproprotein [*Homo sapiens*]	gi|4826762	45861	6.13	28.2	5.9	63	0.011
**5**	Haptoglobin isoform 1 preproprotein [*Homo sapiens*]	gi|4826762	45861	6.13	18.9	5.9	58	0.029
**6**	Haptoglobin isoform 1 preproprotein [*Homo sapiens*]	gi|4826762	45861	6.13	18.1	6.2	75	0.017
**7**	Haptoglobin isoform 1 preproprotein [*Homo sapiens*]	gi|4826762	45861	6.13	18.2	5.5	59	0.028
**8**	Transthyretin [*Homo sapiens*]	gi|48145933	15991	5.52	20.2	6.1	94	0.0002
**9**	Transthyretin [*Homo sapiens*]	gi|48145933	15991	5.52	20.2	5.9	115	1.6e-06

**Figure 2:**
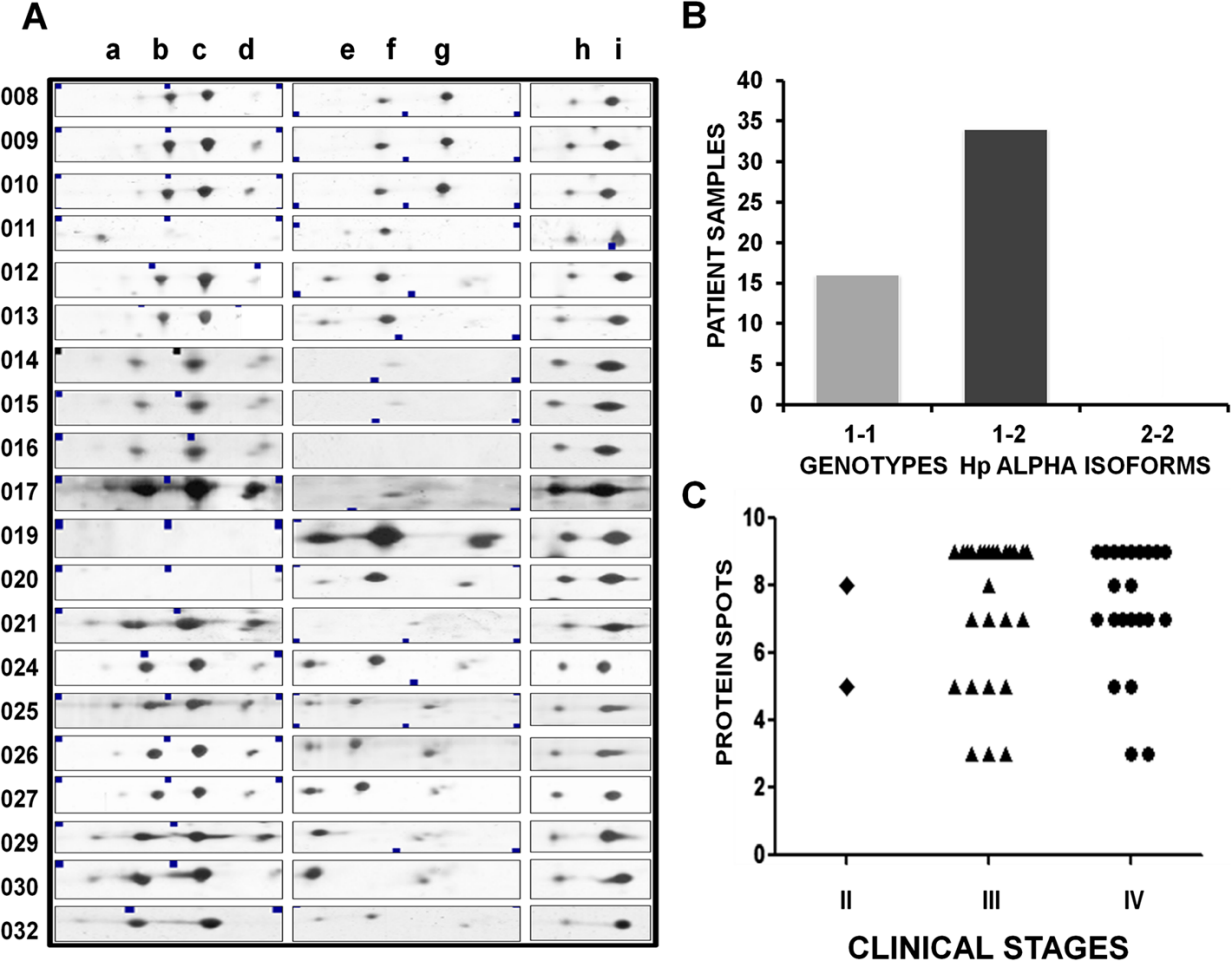
**Phenotypes and genotypes of haptoglobin alpha isoforms in Mexican patients. A)**. Selected area of the protein profiles obtained from 20 different and independent samples, representative of the total 50 samples collected for this study. Protein samples (50 μg) from ascitic fluids were processed for 2D after HAS/IgG depletion. **B)**. Hp alpha genotypes (1–1, 1–2, 2–2) present in all 50 samples were determined and counted. **C)**. Graph showing the number of protein spots detected by 2D in the selected area for each of the 50 patients according to their clinical stage.

We then proceeded to detect protein expression changes among clinical samples. A comparative analysis of the nine spots previously selected, in 20 representative samples of the 50 analyzed is shown (Figure 2A). Results of this analysis showed that Transthyretin isoforms retained a constant expression and concentration pattern in all the analyzed samples. In comparison, Hp alpha showed differential expression patterns and different concentration levels. The molecular weight of the isoforms was of 28 kDa (isoforms a-d) and of 18 kDa (isoforms e-g) (Figure 2A). Human Hp is formed by a tetramer with two subunits, described as α2β2. There are three genotypes of Hp alpha subunit: Hp 1–1, Hp 1–2 and Hp 2–2, which differ in their structure and size. During the ascitic fluid analyses, two genotypes of the alpha subunit were found, one of the seven spots (b spot) was identified as an alpha 2 isoform, and the six remaining spots corresponded to the alpha 1 isoform. With the data obtained by mass spectrometry, we analyzed the presence of Hp alpha genotypes in the ascitic fluids of the patients. The results show that in the Mexican population analyzed in this study, the most prevalent genotype was Hp alpha 1–2 (70%), and the less frequent was the Hp alpha 1–1 genotype (30%). The Hp alpha 2–2 genotype was absent (Figure 2B). The genetic variation of the alpha 1–2 Hp isoform has been associated with the prevalence and clinical evolution of many inflammatory diseases [11].

To establish a probable association between the number of Hp alpha isoforms and the clinical stage of the patients’ illness, a scatter plot graphic was produced. Results suggest that clinical stage IV samples (44% of total samples), correlate with a higher number of spots of this protein; of these, 81.8% had from 7 to 9 spots, whereas only 73% of clinical stage IIIC samples (52% of total samples) had from 7 to 9 spots (Figure 2C).

**Figure 3:**
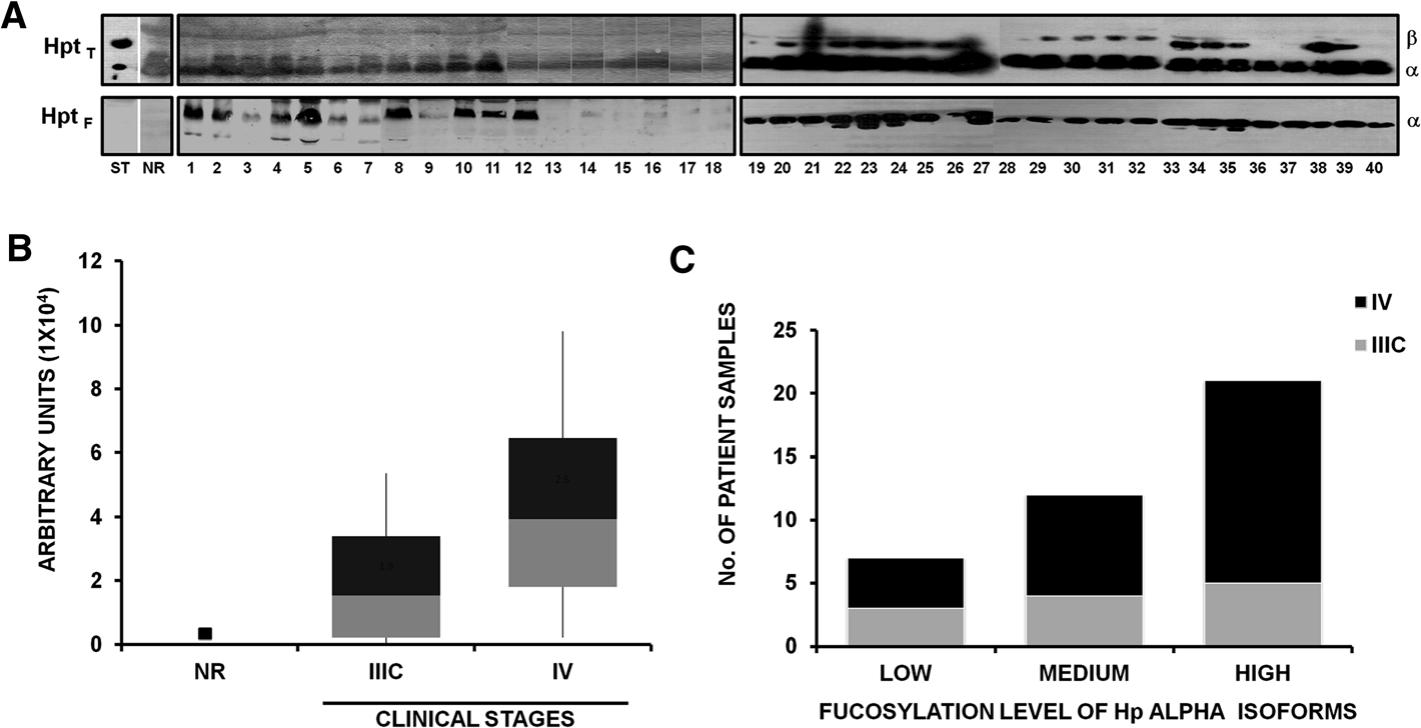
**Fucosylation profile of haptoglobin. A)**. Haptoglobin standard (ST), an ascitic fluid sample non-related with cancer (NR), and 40 ascitic fluid samples from ovarian cancer patients were analyzed for fucosylation of haptoglobin alpha. Protein samples (50 μg) from ascitic fluid free of abundant proteins were processed through a 12.5% SDS-PAGE and transferred to a NCP to incubate with biotinylated-*Aleuria aurantia* lectin (1:1000), specific for fucose residues; variable levels of fucosylation were detected by a colorimetric system using Alkaline Phosphatase-conjugated Streptavidin (1:2000) (lower left panel) or with Horseradish Peroxidase-conjugated Streptavidin (1:1000) (lower right panel). As loading control, the same samples were incubated with an anti-Hp antibody that recognizes the β, α1 and α2 isoforms of Hp to detect total Hp, and a secondary antibody conjugated with Horseradish Peroxidase (1:1000) (upper right panel) or Alkaline Phosphatase-conjugated antibody (1:1000) (upper left panel). **B)**. Graph showing densitometric values of samples’ fucosylation in arbitrary units, comparing clinical stages IIIC and IV vs. a non-cancer related sample. **C)**. Fucosylation levels (low, medium or high) of samples, according to clinical stages.

Several studies have identified Hp, particularly the glycosylated forms produced by fucosylation, whose levels are increased in cells and serum from ovarian cancer patients [23-25]. To see whether Hp alpha isoforms, present in ascitic fluids, were fucosylated, specific-ligand binding assays using biotinylated *Aleuria aurantia* lectin (AAL) were performed. The presence of total Hp was monitored by the western blot method using a commercial anti-Hp monoclonal antibody. Figure 3A shows the profiles of Hp_T_ (beta and two alpha isoforms) in ascitic fluids from 40 patients. This analysis included an ascitic fluid sample not related to cancer (NR), as well as a commercial Hp standard (ST). It is noteworthy that a differential degree of fucosylation (Hp_F_) is appreciated in the alpha chain of Hp, shown in the bottom panels of both filters, which highlights in some samples, the presence of two fucosylated alpha chains (18 and 28 kDa). The absence of fucosylation of the Hp standard (purified from healthy human sera), and that from a non-cancer related sample from a hepatic cirrhosis case, suggest that this post-translational modification is only present in Hp from ascitic fluid samples derived from ovarian cancer patients.

The differential level of Hp fucosylation was analyzed in a scatter plot graphic using densitometry and clinical staging. The results show a trend of higher fucosylation of Hp alpha in stage IV cases (Figure 3B).

Fucosylation levels determined by densitometric analysis were classified as high, medium, or low. It was then possible to establish the relationship between the levels of fucosylation with respect to the clinical stage of the patients analyzed. Results show that in samples from patients at stage IV (68.4%), 53.8% were highly fucosylated. In comparison, samples from patients at stage IIIC (31.6%) showed a more heterogeneous pattern of fucosylation, where from these, 25% had low fucosylation levels, 33.3% had medium level and 41.7% showed high levels of fucosylation (Figure 3C). To analyze the statistical significance of the differences in fucosylation of the Hp alpha isoform and its possible correlation with the clinical stage of each patient, a Spearman correlation between these criteria was produced. A directly proportional relationship, statistically significant, was found between fucosylation levels and advanced clinical stage of the patients (*r*_*s*_ = 0.6664, *p* ≤ 0.05). This established that the clinical stage IV is associated with a higher level of fucosylation of Hp alpha.

**Figure 4:**
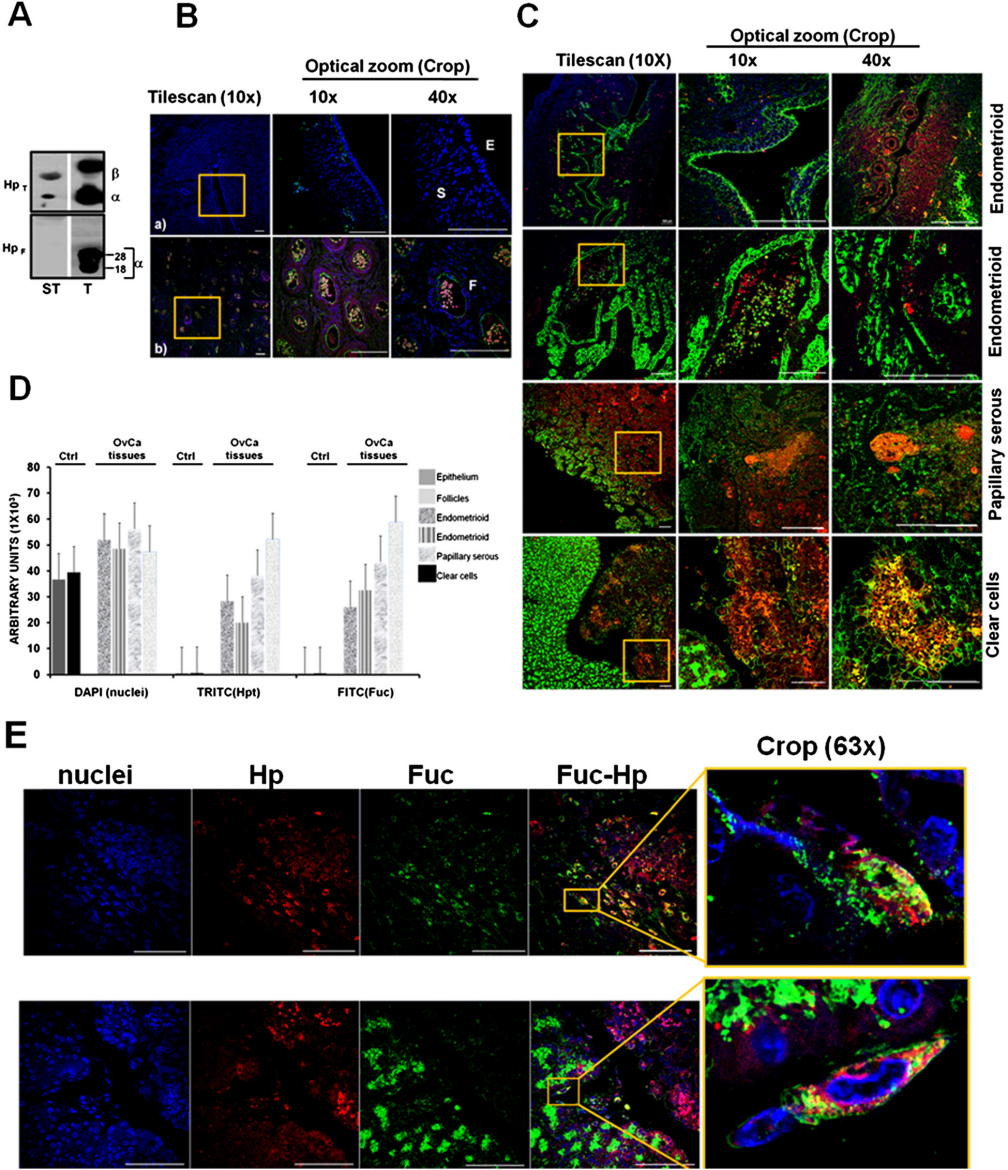
**Immunolocalization of haptoglobin and its fucosylation state in ovarian carcinoma. A)**. Detection of total (Hp_T_) and fucosylated Hp (Hp_F_) in a Hp commercial standard (ST) and a tissue extract from an ovarian tumor developed in Nu/Nu mice (T) by Western blot and lectin binding assays (as described in figure 3). **B, C, E)**. Immunolocalization of Hp, fucosylated Hp and fucosylation. Tissue nuclei were stained with DAPI (blue) to help define the tissues’ structures; Hp was stained with a primary anti-Hp antibody and was developed with a secondary TRITC coupled antibody (red); fucosylation was detected by biotinylated-*Aleuria aurantia* lectin followed by FITC coupled streptavidin (green). All reagents were used at a 1:100 dilution and the scale bar = 100 μm. **B)**.Histo-immunofluorescence of stromal and epithelial (a) and germinal (b) areas of cancer-free ovarian tissue slices. The analysis of tile scan shows a tridimensional image generated by 25 fields’ caption (left panels); from the tile scan, an area was selected (yellow square) and optical zooms were done twice (10X and 40X). Epithelium (E), stroma (S), and follicles (F). **C)**. Tumor tissue sections from four different patients. Fucosylation of Hp was analyzed in broad zones (5x5) of tissues as a tile-scan analysis. A representative region (yellow box) of each tissue was selected to detect fucosylated Hp in optical zooms. **D)**. Graph showing a semi-quantitative analysis of nuclei, Hp and fucosylation content, considering an average area. This analysis was performed with the Software Zen 2011 (blue edition, Carl Zeiss) determining the mean fluorescence intensity for each molecule of interest given in arbitrary units. **E)**. Immunolocalization of fucosylated Hp in individual cells in an optical zoom from a selected region of a tumor tissue section; the crop section analyzed (yellow box) clearly shows the cytoplasmic distribution of fucosylated Hp with approximately 80% of co-localization found in each selected cell.

The presence of fucosylated Hp alpha in cancer tissues has been described previously [26]. However, there are still two unanswered questions regarding the fucosylation of Hp: one is whether cancer cells themselves produce fucosylated Hp; the other one is whether cell transformation produces a factor which induces the production of fucosylated Hp in the liver [27]. To answer the first question, western blotting assays and specific ligand binding assays with AAL were carried out to establish if fucosylated Hp is produced *in situ* in cancer tissues. The presence of fucosylated Hp was detected in tumors developed in immunodeficient mice, using the ovarian cancer cell line SKOV-3 (Figure 4A). To corroborate the presence of fucosylated Hp in human ovarian cancer tissues, immunohistological assays were performed by confocal microscopy, where a tile scan analysis allowed evaluation of the signals identified in broad zones of the tissues. The distribution patterns of Hp (red), fucosylation (green) and fucosylated Hp (yellow) were observed. Results in Figure 4B show the presence of fucosylated Hp in cancer-free ovarian tissue; this glycoprotein was located mainly inside clearly-defined follicles at any developmental stage (F), but not in superficial epithelium (E) or cortical stroma (S). Basal fucosylation (green) levels were found in stroma, but none in the epithelium. Figure 4C shows the results obtained with three different histotypes of ovarian cancer (papillary serous, endometrioid and clear cell). An over-expression of fucosylation, most probably of a wide variety of proteins broadly distributed in all tissues in comparison with cancer- free tissues, not only in surface epithelium but also in the stroma, was observed. A wide distribution of patch-like patterns in internal and cortical stroma; and a clearly delimited pattern present in membranes of the surface epithelium were found. In areas of highly differentiated tissue, the level and distribution of fucosylated Hp alpha was limited, with a co-localization ratio not higher than 20%. However, in undifferentiated zones of the tissues where certain disaggregated cells can be found, the presence of fucosylated Hp was higher with a colocalization ratio of 60%. Non-fucosylated Hp was clearly seen in all cancer tissues; however, its presence was minimal in comparison with fucosylation levels. Figure 4D shows histograms of fluorescence intensity. These results show that three different processes are occurring in ovarian cancer in comparison to cancer-free tissues: higher levels of fucosylation, non-fucosylated and fucosylated Hp (Figure 4D). Optical zoom images carried out with two of the biopsies allowed us to define more precisely the subcellular localization of fucosylated Hp in tumor cells (Figure 4E). The left panels show the presence of cells which are part of the tumor tissue, expressing intracytoplasmic fucosylated Hp. It can also be appreciated that there is expression of another kind, yet to be identified, of highly fucosylated proteins in cell membranes.

## Discussion

Over more than three decades of clinical use, CA-125 has proven to be one of the most useful tumor markers in cancer medicine; the major clinical utility of this serum marker is in following the clinical course and the evaluation of the effectiveness of new antineoplastic agents against ovarian cancer [28]. However, it is associated with a high false-positive rate among women with benign gynaecological conditions such as endometriosis [15]. Furthermore, CA125 has very low sensitivity in identifying patients with early-stage ovarian cancer [29].

HE4 (human epididymal secretory protein E4; WAP four-disulphide core domain protein 2) alone or in combination with CA125 [30,31], are the only two biomarkers US FDA approved for monitoring of disease recurrence or progression, but not for screening. Recently, there has been a resurgence of efforts to identify ovarian cancer biomarkers. Broadly studied are mesothelin [14]; transthyretin [32]; ApoA1 [33]; IL-6 and IL-8 [34] and serum amyloid A [35] for using in initial detection, staging, disease prognosis, or VEGF studied as a possible molecular therapeutic target. BRCA1 and BRCA2 have been used, alone or in combination, for an individualized clinical management of patients [36,37], to establish the sensitivity or resistance to chemotherapy. Therefore, the search for new biomarkers useful in the early diagnosis of ovarian cancer continues.

Acute-phase proteins are synthesized by the liver as a nonspecific response to inflammation, with the primary function of modulating the inflammatory response to avoid tissue damage or infection [38]. Haptoglobin, an acute-phase glycoprotein, is produced mainly in the liver where it is released into the blood circulation. This protein also participates in the formation of new blood vessels and vascular remodeling [26,39-42].

The presence of Hp in patients’ sera with ovarian cancer and its possible use as a biomarker for the diagnosis of this disease has been suggested previously [43]. Moreover, using ascitic fluid from ovarian cancer patients and mass spectrometry, Faça et al., [44] suggested that the alpha subunit of Hp could be a good biomarker candidate. Although the correlation between Hp expression and ovarian carcinoma has been widely reported [45,46], this is the first study in which it is possible to distinguish differential alpha subunit expression together with differential levels of fucosylation. Our results suggest that the differential expression pattern of isoforms of Hp alpha and the conserved expression profile of Transthyretin might correspond to a signature for each patient, and whose pattern could be representative of the ovarian cancer ascitic fluid stage. Moreover, the genetic prevalence of Hp 1–2, which has been associated with other pathologies of clinical importance, allowed us to establish a significant relationship between the patient Hp alpha pattern and the clinical progress of the disease [11].

Post-traductional modifications of proteins may play important roles in many types of cancer [47]. Increased levels of fucosylation have been reported in a number of pathological conditions, including several cancer types (cervical, gastrointestinal, pancreatic, and breast and ovarian cancer) [26,48-53]. The fucosylation profiles from the alpha subunit of Hp showed that those patients with most advanced stages of cancer expressed the highest levels of fucosylation, suggesting a possible role for fucosylated Hp during the progress of ovarian carcinoma. Our results are in agreement with those described previously, which correlated fucosylation levels of Hp with disease progression in breast cancer [51], gastrointestinal cancer [49], and in adenocarcinoma of the uterus [54]. However, the examination of a greater number of clinical cases is required to confirm this hypothesis.

Since ascites occurs in patients only at advanced stages of the disease, it is important to assess the expression profile of fucosylated Hp in tumor tissues. Differences in the expression of Hp and fucosylated Hp in those tissues in comparison with cancer-free samples were evident. On the one hand, we show for the first time the presence of fucosylated Hp within cancer-free human ovarian follicles. The localization of Hp in healthy ovaries and the possibility that it may play a role in human reproduction has been analyzed; clinical findings depicted the Hp expression pattern in various organs of the reproductive system: the rat ovary [55], the mouse uterus and ovary [56] and the rabbit uterus [57]. The presence of Hp has been reported in human reproductive tissues, as uterus [58] and follicular fluid [59], as a measure of ovary quality.

The differential distribution of fucosylation in tissues of at least three of the five histological types analyzed, in comparison with basal fucosylation observed in cancer-free tissue, suggest two possible directions for further investigation: the involvement of fucosyltransferases overexpression in the process of fucosylation and the identification of highly fucosylated proteins in the initial stages of ovarian cancer.

Even though the liver is considered as the major site for Hp expression, the stimulation with bacterial lipopolysaccharide (LPS) has demonstrated that Hp synthesis can be induced not only in the liver but also in other tissues, including lung, skin, spleen, and kidney [60]. Elevation of this peptide has been observed in infections, inflammations, and various malignant diseases, including lung and bladder cancers [61], leukemia [62], breast cancer [63], malignant lymphoma [64,65], urogenital tumors [66], esophageal squamous cell carcinoma [67] and serum and ascitic fluid of patients with ovarian cancer [68] in comparison to healthy tissue. In this work we provide evidence to support that in addition to the liver as a source of Hp, there is an important participation of tumoral cells in fucosylated Hp production, mainly in tumor zones with higher disintegration and cellular de-differentiation, suggesting a possible role for Hp in the migration of cancer cells.

Moreover, the potential of this work lies in the fact that we are proposing not just haptoglobin as biomarker, but to consider also the number of differential isoforms of alpha haptoglobin and their level of fucosylation present in the tumor tissue. This opens the possibility to determine if these isoforms, glycated or not, could be detected in serum, and if the fucosylation of tissular haptoglobin could help to distinguish between a healthy and a malignant tissue through the analysis of biopsies newly obtained from patients in hope of some diagnosis, whether it be in ovarian cancer or another. Still, there are some limitations within the study, mainly because the number of analyzed samples does not allow setting haptoglobin, in a definitive way, as a specific biomarker for ovarian cancer or its early detection, because it would be necessary to analyze samples from stages I and II. Based on the results obtained, at this moment it is difficult to assign clinical implications for haptoglobin detection; however the difference in the levels of fucosylation between healthy and malignant tissues was very clear, which means that there must be many other fucosylated proteins in malignant tissues.

The use of a glycomic approach, has allowed the identification of a number of tumor marker candidates, finding that fucosylated haptoglobin in sera could be a possible tumor marker for several kinds of cancer types. A detection kit for pancreatic cancer via a sandwich enzyme-linked immune sorbent assay using *Aleuria aurantia* lectin and the Fab portion of anti-haptoglobin antibody has been reported [69]. Positive rates of fucosylated haptoglobin with this method were significantly higher in patients of stage IV pancreatic cancer, compared with other clinical stages.

At this moment, we would not dare to recommend patients to undergo tests to determine if there is fucoyslated Haptoglobin alpha isoforms in ascitic fluids, but indeed, this is a very good perspective for this work, i.e., to establish a simple, low-cost laboratory test, while satisfying the requirements for sensitivity and specificity required to convert to fucosylated haptoglobin in a reliable biomarker together with CA125 or computerized axial tomography to evaluate the ability of complete resection, or to follow if there is response to drug therapy. Without leaving aside that the process of fucosylation could also become a very important early marker of cellular transformation.

## Conclusion

Based on the above results, we propose that increased numbers of highly fucosylated Hp alpha isoforms in ascitic fluids as well as the presence of fucosylated Hp in tumor tissues of Mexican ovarian cancer patients are associated with advanced stages of the disease; and reinforce the potential of fucosylated Hp alpha isoforms to be used as biomarkers for disease progression.

## Competing interests

The authors declare that they have no competing interests.

## Authors’ contributions

This work was carried out through the collaboration of all the authors. TRP and GCO were responsible of the research design; GRD and CLD were involved in the design of the protocol to submit to the Ethics and Scientific Committees to get their approval, in the diagnosis, selection, and collection of biological samples. EGS and HOM were involved in mass spectrometry protocols. GCO, HRVI and OTJC carried out the experiments, and together with TRP and VPJC participated in the results interpretation and drafting of the manuscript. All authors had read and approved the final manuscript.

## Supplementary Material

Additional file 1: Figure S1 Tridimensional and densitometric analysis of the proteins with differential expression pattern. A). Representative images of the tridimensional analysis images with Melanie Software, showing the constitutive pattern of spots h and I, and variant of spots a to g, among 20 different samples. B). Comparative densitometric analysis of the content of nine proteins (spots a to i) in twenty independent samples of ascitic fluid by Melanie confirming the reproducibility in the amount of protein loaded in each gel. Click here for file
