# Supplementary figures and images for: Proteomic identification of fucosylated haptoglobin alpha isoforms in ascitic fluids and its localization in ovarian carcinoma tissues from Mexican patients
Source: J Ovarian Res. 2014 Feb 27;7:27. doi: 10.1186/1757-2215-7-27 (PMC3943579; doi:10.1186/1757-2215-7-27)

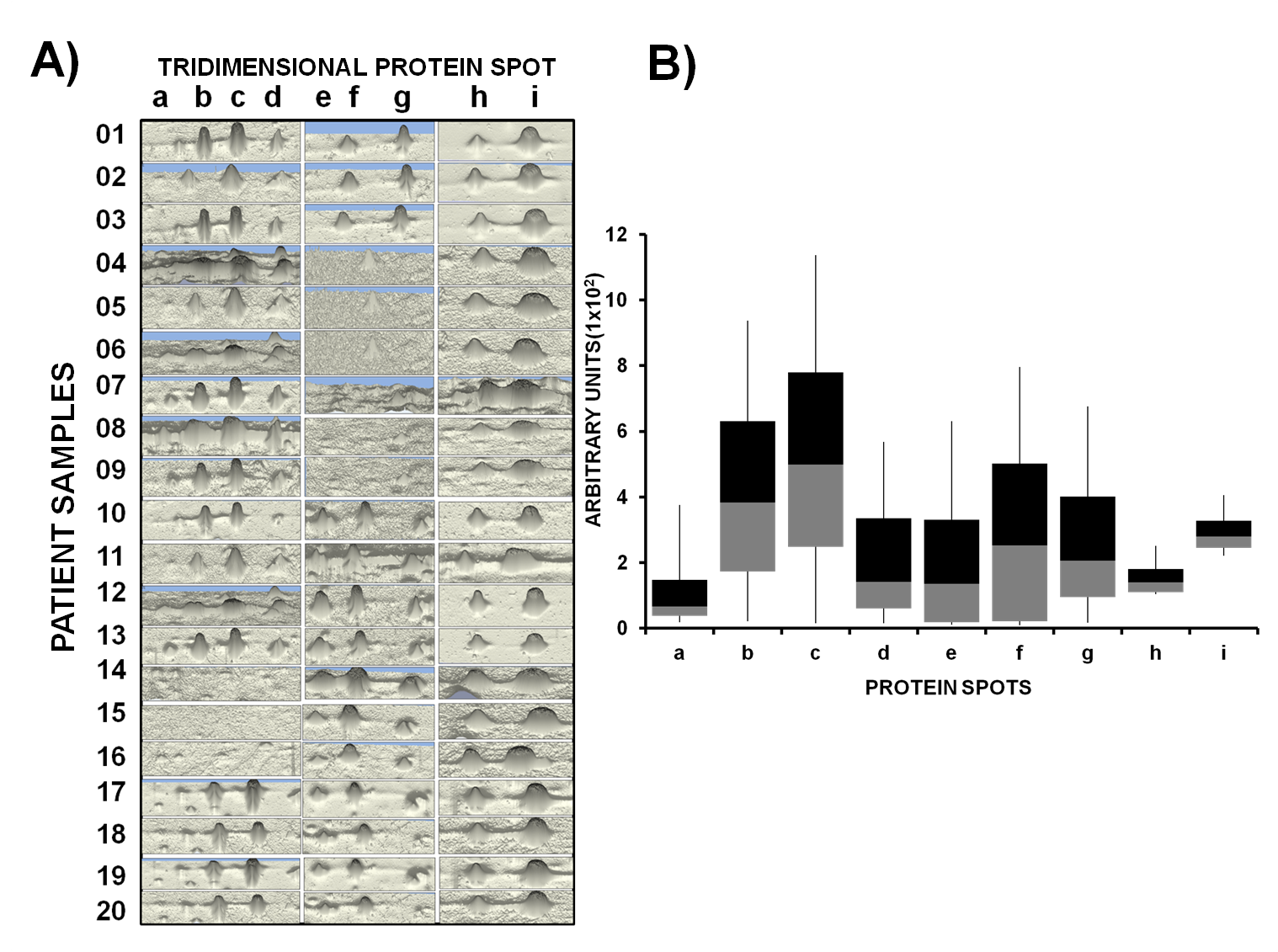

Supplement: Additional file 1: Figure S1 — Tridimensional and densitometric analysis of the proteins with differential expression pattern. A). Representative images of the tridimensional analysis images with Melanie Software, showing the constitutive pattern of spots h and I, and variant of spots a to g, among 20 different samples. B). Comparative densitometric analysis of the content of nine proteins (spots a to i) in twenty independent samples of ascitic fluid by Melanie confirming the reproducibility in the amount of protein loaded in each gel. [file 1757-2215-7-27-S1.tiff]
